# MiR-1a-3p/Fcgr4-dependent osteoclast activation regulates pathological bone loss

**DOI:** 10.3389/fimmu.2026.1828877

**Published:** 2026-05-25

**Authors:** Jiayao Zhang, Yun Zhai, Liang He, Yunping Song, Mingxuan Lu, Xuerui Xiang, Jiehong Huang, Jinyin Huang, Weiqing Tian, Yue Zhao, Shuxian Lin, Weicai Liu

**Affiliations:** 1 Shanghai Engineering Research Center of Tooth Restoration and Regeneration & Tongji Research Institute of Stomatology & Department of Prosthodontics, Shanghai Tongji Stomatological Hospital and Dental School, Tongji University, Shanghai, China; 2 Department of Orthopedics, Shanghai Key Laboratory for Prevention and Treatment of Bone and Joint Diseases, Shanghai Institute of Traumatology and Orthopedics, Ruijin Hospital, Shanghai Jiaotong University School of Medicine, Shanghai, China; 3 Department of Neurology and Neurological Rehabilitation, Shanghai Disabled Persons’ Federation Key Laboratory of Intelligent Rehabilitation Assistive Devices and Technologies, Yangzhi Rehabilitation Hospital (Shanghai Sunshine Rehabilitation Center), School of Medicine, Tongji University, Shanghai, China; 4 Institutional Center for Shared Technologies and Facilities of SIII, CAS, Shanghai, China

**Keywords:** FcγRIV, lumbar spine, miR-1a-3p, osteoclast, osteoimmunology

## Abstract

**Introduction:**

Osteoporosis is a systemic metabolic disease characterized by disrupted homeostasis between osteoclast-mediated bone resorption and osteoblast-mediated bone formation. Accumulating evidence indicates that chronic systemic pathological states can exert sustained effects on osteo-immune homeostasis. However, how these disturbances promote immune-mediated osteoclast dysregulation remains unclear.

**Methods:**

Candidate miRNAs targeting *Fcgr4* were identified using bioinformatic prediction tools, and the direct interaction between miR-1a-3p and *Fcgr4* was validated by dual-luciferase reporter assay. RAW264.7 cells were transfected with miR-1a-3p mimics or inhibitors to assess the effects of miR-1a-3p on osteoclast, which was evaluated by tartrate-resistant acid phosphatase (TRAP) staining, RT-qPCR, and Western blotting. miR-1a-3p expression was further analyzed in human osteoporosis cohorts and animal models of bone loss. Furthermore, to investigate whether systemic psychological stress—a chronic pathological state with sustained immunoregulatory consequences—regulates this axis, a chronic unpredictable mild stress (CUMS) model was established to examine stress-associated regulation of this axis. miR-1a-3p expression were detected by qRT-PCR, and FcγRIV–SYK–NFATc1 pathway activation was assessed by immunofluorescence staining, qRT-PCR, and Western blot.

**Results:**

We identified *Fcgr4* as a direct target of miR-1a-3p and found that miR-1a-3p overexpression significantly suppressed osteoclast activity by inhibiting *Fcgr4*-dependent signaling. Consistent with this regulatory relationship, miR-1a-3p expression was significantly reduced in both human osteoporosis cohorts and animal models of bone loss. In the CUMS model, decreased miR-1a-3p expression was accompanied by increased *Fcgr4* expression, activation of the FcγRIV–SYK–NFATc1 signaling pathway, enhanced osteoclast activity, and osteoporotic bone loss.

**Discussion:**

These findings support a role for the miR-1a-3p–*Fcgr4* axis in linking systemic pathological changes to immune-mediated osteoclast dysregulation, providing a mechanistic framework for pathological bone loss and a basis for understanding immune-bone interactions in chronic systemic diseases.

## 1 Introduction

Osteoporosis is generally understood as a bone metabolism disorder caused by an imbalance between osteoblast‐mediated bone formation and osteoclast‐mediated bone resorption (1–4). Therefore, current therapeutic strategies mainly target canonical intraosseous signaling pathways, such as the RANKL–RANK axis, to suppress bone resorption or enhance bone formation. However, these approaches often provide only partial preservation of bone mass (5, 6). One possible reason is that they focus predominantly on bone-intrinsic regulation while underappreciating the sustained effects of systemic pathological states—including aging, metabolic disorders, and chronic inflammation—on bone homeostasis through altered inter‐organ communication (4, 7). This limitation may contribute to heterogeneity of therapeutic responses among patients with osteoporosis and increase susceptibility to rebound bone loss after treatment withdrawal (8). These observations further suggest that, despite their distinct origins, diverse systemic pathological insults may ultimately act through common downstream pathways to drive persistent bone loss. Identifying such shared mechanisms may therefore provide a more integrated perspective on persistent bone loss across diverse etiologies.

In this context, osteoclasts are of particular interest because they originate from the monocyte–macrophage lineage and retain prominent immunological features throughout their differentiation and functional activation. This enables them to sense changes in systemic immune status and convert into altered bone resorptive activity (9, 10). Thus, osteoclasts occupy an important position at the interface between immune regulation and skeletal remodeling. Among these immune‐related receptors expressed by osteoclasts, receptors such as receptor activator of nuclear factor-κB (RANK) and colony stimulating factor 1 receptor (CSF1R), primarily determine lineage commitment and differentiation (3, 11), whereas others, including DNAX-activating protein of 12 kDa (DAP12)‐associated receptors, function as immunoreceptors that could amplify activation signals (12). Although both receptor classes are essential for osteoclast differentiation and maturation, they are highly conserved components of the osteoclastogenic program. By contrast, Fc gamma receptors (FcγRs) are activating transmembrane immunoreceptors that recognize immunoglobulin G (IgG) immune complexes and may therefore provide a more direct mechanism by which osteoclasts sense systemic immune status (13). In addition, FcγRs signal through the ITAM–SYK–NFATc1 pathway, thereby directly regulating osteoclast activation and bone resorptive function (14, 15). Notably, FcγR expression is largely enriched in macrophages and osteoclasts, conferring a degree of cellular specificity that strengthens its relevance as a mechanistic target. The FcγR family comprises multiple subtypes, including FcγRI, FcγRII, FcγRIII, and FcγRIV. Among these, FcγRIV, encoded by *Fcgr4* in mice, is particularly notable because it exhibits heightened sensitivity (16) under inflammatory conditions and preferentially responds to the most potent IgG subclasses (13). Consistent with this, FcγRIV activation has been shown to promote osteoclastogenesis and bone resorption (16, 17), supporting the idea that it may serve as a molecular link between systemic immune activation and osteoclast-mediated skeletal degradation. However, whether this pathway contributes, at least in part, to bone loss under chronic pathological conditions remains unclear.

Nevertheless, although elevated IgG levels and immune complexes can activate FcγRIV and promote osteoclast activation, antibody abundance is tightly regulated by immune homeostasis and generally fluctuates rather than remaining persistently elevated (18). Thus, IgG–FcγRIV signaling alone is therefore unlikely to provide a sufficient and sustained drive for chronic osteoclast overactivation. Instead, chronic pathological states may act through more durable regulatory mechanisms (19). In this context, microRNAs (miRNAs) are particularly relevant because their relative stability in circulation and capacity for intertissue transfer allow them to integrate persistent systemic states and mediate durable regulatory effects (20, 21). Consistent with this, multiple miRNAs are dysregulated across primary and secondary osteoporosis and exert functional effects on osteoclast. For instance, miR-144-5p is reported to be elevated in type 2 diabetes and to promote osteoclast differentiation via the RANKL–RANK–OPG pathway (22), whereas separate studies have shown that miR-124-3p is reduced in chronic kidney disease (23) and inhibits osteoclastogenesis through nuclear factor of activated T cells 1 (NFATc1) (24). Together, these observations support a miRNA-mediated regulatory architecture through which chronic systemic disturbances may influence osteoclast immune responsiveness by modulating immunoreceptor expression and downstream signaling, thereby contributing to pathological bone resorption. Within this framework, identifying key miRNAs that regulate FcγRIV becomes essential for understanding how systemic cues are persistently translated into excessive osteoclast activation and impaired bone mass.

In the present study, we demonstrate that miR-1a-3p directly targets *Fcgr4* and negatively regulates osteoclast differentiation. We further show that miR-1a-3p expression is significantly reduced in osteoporosis models, coinciding with enhanced osteoclast activation. Notably, our results suggest that miR-1a-3p is responsive to psychological stress, a systemic pathological state with sustained immunoregulatory consequences, further indicating that its regulation is shaped not only by the local bone microenvironment but also by broader systemic cues. Collectively, our findings identify a previously unrecognized miR-1a-3p–Fcgr4 regulatory axis through which chronic systemic disturbances may relieve restraint on immune receptor signaling and thereby sustain osteoclast overactivation and pathological bone loss. This work provides mechanistic insight into how systemic signals can be translated into osteoclast dysregulation through miRNA-dependent control of an activating immunoreceptor pathway, and suggests that targeting this axis may offer a complementary strategy for osteoporosis intervention beyond conventional bone-intrinsic approaches.

## 2 Materials and methods

### 2.1 Animals

C57BL/6 male mice, aged 6 weeks, were obtained from the Shanghai Model Organisms Center, Inc. All animal experiments were conducted with the approval of the Animal Ethics Committee of Tongji University, Shanghai, China (Number: TJ-HB-LAC-2023-39). Animals were housed in standard cages (six mice in each cage) under standard conditions of constant temperature (22-24 °C) and a 12-hour light/dark cycle, with free access to food and water, for 1 week prior to the experiment at the Hubei Laboratory Animal Center, Tongji University.

### 2.2 Prediction of miRNAs targeting *Fcgr4*

To identify candidate miRNAs targeting *Fcgr4* with potential relevance to osteoclast biology, we first screened *Fcgr4*-targeting miRNAs using the DIANA platform (https://dianalab.e-ce.uth.gr/) and TargetScan (https://www.targetscan.org/). Candidate miRNAs with high prediction scores were then cross-referenced with miRNAs reported to be associated with osteoclast differentiation. The tissue expression patterns of the shortlisted miRNAs were further examined using miRNA Tissue Atlas 2025 (https://web.ccb.uni-saarland.de/mirnatissueatlas_2025), and miR-1a-3p was selected for subsequent analyses.

### 2.3 3’UTR luciferase reporter assay

A fragment of the *Fcgr4* 3′ UTR containing the wild-type (WT) or mutant (Mut) sequence, which was predicted to contain two binding sites for miR-1a-3p by the TargetScan database (http://www.targetscan.org), was inserted into the pMIR-REPORT vector. HEK293 cells were co-transfected with either *Fcgr4*-WT or *Fcgr4*-Mut vector and either miR-1a-3p mimic or mimic-NC (negative control) using Lipofectamine 2000 (Invitrogen, USA; 11668019). Luciferase activity was determined at 48h post-transfection using the Dual-Glo Luciferase Assay System (Promega, Madison, WI; E2940) in accordance with the manufacturer’s instructions. Data were normalized by dividing firefly luciferase activity with that of Renilla luciferase enzyme activity.

### 2.4 Cell culture and transfection

The RAW 264.7 cells were provided from Dr. Shengbing Yang at the Shanghai Key Laboratory of Orthopaedic Implants, Department of Orthopaedic Surgery, Shanghai Ninth People’s Hospital, Shanghai Jiao Tong University School of Medicine, Shanghai, China and cultured using α-MEM basic medium supplemented with 10 % fetal bovine serum (FBS; Sigma-Aldrich, St. Louis, MO, USA; F8318) and 1 % penicillin–streptomycin at 37°C in a humidified atmosphere with 5 % CO_2_. Receptor activator of nuclear factor-κB Ligand (RANKL) (R&D Systems, USA; 462-TEC) at a concentration of 50nM was used to induce osteoclastic differentiation for three days. In addition, once the cells had reached 50% confluence, they were co-transfected with either mimic-miR-1a-3p or mimic-NC and *Fcgr4* overexpression (*Fcgr4*-OE) plasmids (QianMo Biotechnology Company, Shanghai, China) using Fugene HD (Promega, Madison, WI, USA; E2311). The transfection ratio (Fugene HD: mimic) was 1.2:0.7(uL). Trap staining, pit formation assays and phalloidin staining were employed to detect osteoclasts derived from RAW 264.7 cells.

### 2.5 Quantitative real-time PCR analysis

Total RNA was extracted from tissues or cells using the RNAiso Plus reagent (Takara Biotechnology, Japan; 9109), and the RNA concentration was assessed using a NanoDrop One spectrophotometer. Complementary DNA (cDNA) was synthesized using a PrimeScript™ RT Master Mix Kit (Takara Biotechnology, Japan; RR036A). For miRNAs, miDETECT A Track™ miRNA qRT-PCR Starter Kit (RIBOBIO, Guangzhou, China; C10712-1) was employed. qRT-PCR was performed on CFX Opus 96 (Bio-Rad Laboratories, Hercules, CA, USA) using SYBR Green Realtime PCR Master Mix (Yeasen, Shanghai, China; 11201ES08). The expression levels of the target gene were calculated by the comparative Ct (2^−ΔΔCT^) method using *Gadph* or U6 for normalization. The primer sequences were as follows:

**Table:** .

GENE	5’-3’
mouse-*Fcgr4*-F	TGGTGAACCTAGACCCCAAG
mouse-*Fcgr4*-R	GTGGGATGAGGCTTTCGTTA
mouse-*Acp5*-F	GACAAGAGGTTCCAGGAGACC
mouse-*Acp5*-R	GGGCTGGGGAAGTTCCAG
mouse-*Ctsk*-F	GAAGCAGTATAACAGCAAGGTGGAT
mouse-*Ctsk*-R	TGTCTCCCAAGTGGTTCATGG
mouse-*Ca2*-F	GCTGCAGAGCTTCACTTGGT
mouse-*Ca2*-R	AAACAGCCAATCCATCCGGT
mouse-*Mmp9*-F	GGAACTCACACGACATCTTCCA
mouse-*Mmp9*-R	GAAACTCACACGCCAGAAGAATTT
mouse-*Oscar*-F	TGGTCATCAGTTTCGAAGGTTCT
mouse-*Oscar*-R	CAGCCCCAAACGGATGAG
mouse-*Nfatc1*-F	ATGCGAGCCATCATCGA
mouse-*Nfatc1*-R	GGGATGTGAACTCGGAAGAC
mouse-*Syk*-F	CTACCTGCTACGCCAGAGC
mouse-*Syk*-R	GCCATTAAGTTCCCTCTCGATG
mouse-*Gapdh*-F	AGGTCGGTGTGAACGGATTTG
mouse-*Gapdh*-R	GGGGTCGTTGATGGCAACA
mmu-miR-1a-3p-F	UGGAAUGUAAAGAAGUAUGUAU
mouse-*Ocn*-F	CTGACCTCACAGATgCCAAGC
mouse-*Ocn*-R	TGGTCTGATAGCTCGTCACAAG
mouse-*Opn*-F	AGCAAGAAACTCTTCCAAGCAA
mouse-*Opn*-R	GTGAGATTCGTCAGATTCATCCG
mouse-*Alp*-F	CCAACTCTTTTGTGCCAGAGA
mouse-*Alp*-R	GGCTACATTGGTGTTGAGCTTTT
mouse-*Runx2*-F	CCTTTACCTACACCCCGCCA
mouse-*Runx2*-R	GGATGCTGACGAAGTACCAT
mouse-*Dmp1*-F	CATTCTCCTTGTGTTCCTTTGGG
mouse-*Dmp1*-R	TGTGGTCACTATTTGCCTGTC

### 2.6 Analysis of clinical and animal experimental data on osteoporosis

Publicly available datasets used in this study were from the NCBI Gene Expression Omnibus (GEO) under accession numbers GSE93883 (clinical dataset) and GSE221729 (animal experimental data) (25). Differential expression analysis was performed according to the data type of each dataset. Specifically, GSE93883 was analyzed using the limma package (3.58.1) (26) based on the processed normalized expression matrix, whereas GSE221729 was analyzed using the DESeq2 package (v1.34.0) (27) based on the raw count matrix. miRNAs with a fold change ≥ 1.3 and an adjusted *p* value < 0.05 were considered significantly differentially expressed and were displayed in the volcano plots.

### 2.7 The chronic unpredictable mild stress model establishment

After one week of acclimatization, 16 mice were randomly divided into a control group (CON, n=8) and an experimental group (CUMS, n=8). Mice in the CUMS group received random stimulation each day. Stimulations included: the cage being tilted at 45°for 7 h, wet bedding for 24 h, forced swimming for 5 min, restraint in a 50 mL tube for 6 h, tail pinch for 1 min, day/night reversal for 24 h, food deprivation for 24 h, and water deprivation for 24 h. In order to prevent the animals from being able to predict the occurrence of stimulation, it was ensured that the same stimulation would not occur for two days. The protocol lasted 6 weeks, while control mice were housed in groups under normal conditions with daily handling. All animals were weighed once a week at 15:30-16:30.

### 2.8 Behavioral tests

#### 2.8.1 Open field test

The OFT is used to measure physical condition and anxiety-like behavior in a brightly lit open area. The OFT chamber (40 cm × 40 cm × 40 cm) was homemade in the laboratory. Mice were gently placed in the center of the arena and allowed to explore freely for 10 min. The time and distance traveled in the central area (20 cm × 20 cm) were recorded and calculated. The chamber was cleaned with 75% alcohol after each mouse to prevent the odor of the previous mouse from influencing the OFT results.

#### 2.8.2 Tail suspension test

In a dimly lit and quiet room, the animals were suspended 50 cm off the ground for 6 min, using tape placed 1 cm from the tip of the tail. The immobility time during the last 4 min was calculated.

#### 2.8.3 Sucrose preference test

Before the test, each mouse was housed in a single cage and given two bottles of drinking water for 24 h, and then given one bottle of 1% sucrose solution (w/v) and one bottle of drinking water for 24h, followed by 24 h of water and food deprivation. During the test, the mice were offered two bottles of 1% sucrose solution (w/v) or drinking water, and the bottles were swapped mid-test. The bottles were weighed before and during the test. After calculating the consumption of water and sucrose solution, the formula was applied for determining the sucrose preference: sucrose preference = [(sucrose consumption)/(sucrose consumption + water consumption)] × 100%.

### 2.9 Micro-CT analysis

At the end of behavioral tests, the third lumbar vertebrae, femurs and tibias were dissected from each group of mice, fixed in 4% paraformaldehyde for 48 h, and stored in 0.4% paraformaldehyde at 4°C until scanning. The lumbar vertebrae were subjected to Micro-CT 50 analysis (Scanco Medical, Zurich, Switzerland) with a scan resolution of 10 μm, a voltage of 70 kV, and a current of 200 μA. The region of interest (ROI) for structural morphometry and statistical analysis was trabecular bone of the third lumbar vertebrae, as well as that of femur and tibia underneath the growth plate. The quantification of trabecular bone parameters was conducted through the utilization of specific indices, namely bone mineral density (BMD), bone volume over tissue volume (BV/TV), bone volume (BV), trabecular number (Tb.N), and trabecular separation (Tb.Sp).

### 2.10 Compression test

The intervertebral discs were removed and the posterior arch of each vertebra was resected, and the vertebral endplates were then encapsulated in methyl methacrylate cement to obtain two parallel loading surfaces. The load was applied to the vertebra in the cranio-caudal direction, at a speed of 1 mm/min, at room temperature. The mechanical tests were stopped immediately after the ultimate strength was measured. The maximum load (N), stiffness (N/mm), and elastic energy (N*mm) were calculated from the load-displacement curve.

### 2.11 Histologic analysis

The third lumbar vertebrae were fixed in 4% paraformaldehyde for 48 h, decalcified in 10% ethylene diamine tetraacetic acid (EDTA) for 8 weeks, and embedded in paraffin after graded alcohol dehydration. The samples were then cut into 4-μm thick paraffin sections. Hematoxylin & Eosin (H&E) (Beyotime, China; C0105S) and toluidine blue (Solarbio, China; G3668) were conducted to evaluate histological morphology. Osteoclastic activities were detected by tartrate resistant acid phosphatase (TRAP) staining (Sigma-Aldrich, St. Louis, MO, USA; 387A) and immunohistochemical staining for acid phosphatase 5 (ACP5) (Affinity, China; DF6989). The number of osteoclasts (N. OCs/BS/mm) and the TRAP positive surface of osteoclast (OC. S/B) were calculated. At the level of mechanism validation, immunohistochemical staining for FcγRIV (Sino Biological, China; 50036-R011), Cathepsin K (CTSK) (Santa Cruz Biotechnology, CA, USA; sc-48353), NFATc1 (Santa Cruz Biotechnology, CA, USA; sc-7294), 4’,6-diamidino-2-phenylindole (DAPI) (biofroxx, China; 1155MG010) was used to observe the location and abundance of protein expression.

### 2.12 Bone marrow-derived macrophage cells isolation and cell culture conditions

After euthanizing mice in the CON and CUMS groups following cervical dislocation, the bilateral femurs and tibias were isolated under sterile conditions. The bone ends were cut off, the medullary cavities were flushed with sterile PBS, the bone marrow fluid was collected, and red blood cells were lysed after filtration through a 200-mesh cell strainer. After centrifugation (500 × g, 5 minutes), resuspend the cells in α-MEM basic medium supplemented with 10 % FBS and 1 % penicillin–streptomycin Incubate at 37 °C and 5% CO_2_ for 24 hours. Transfer the supernatant to a new culture dish and add 10 ng/mL M-CSF to allow the BMDMs to adhere. For osteoclast differentiation, BMDMs were plated at 5x10^3^ cells/plate in 96-well plates and cultured in medium containing either recombinant murine M-CSF (10ng/mL) and RANKL (50ng/mL).

### 2.13 Western blot analysis

The tissues and cells were lysed in radio immunoprecipitation assay lysis buffer (Beyotime Biotechnology, China; P0013B) for 30 min. The supernatant was centrifuged at 12,000 rotations per minute (rpm) for 15 min at 4°C. Then, the total protein concentration of the supernatants was assessed using a BCA protein analysis kit (Epizyme, China; ZJ102). The supernatant protein samples were mixed with 5 × loading buffer (Epizyme, China; LT101), metal bath at 98 °C for 3 min and stored at -20 °C. Samples were separated by 10% sodium dodecyl sulfate - polyacrylamide gel electrophoresis (SDS-PAGE) and transferred to 0.45 μm polyvinylidene fluoride. The membrane was blocked with protein-free rapid sealing solution(1x) (Epizyme, China; PS108P) for 40 min and then incubated overnight at 4°C with antibodies against phosphorylated spleen tyrosine kinase (p-SYK) (Affinity, China; AF8404), FcγRIV (Proteinch, China, 33531-1-AP), β-tubulin (Affinity, China, T0023) and β-actin (Affinity, China, T0022). Secondary antibodies were incubated for 1h at room temperature. The membranes were treated with ELC chemiluminescent solution (Biosharp, China; BL520A) for imaging. To control for sampling error, the ratio of band intensities to β-actin was obtained to quantify the relative protein expression levels using imageJ.

### 2.14 Statistical analysis

For comparison between normally distributed data within two experimental groups, unpaired two-tailed Student’s *t*-test were used. For normally distributed data with three or more groups, one-way ANOVA with *post-hoc* Tukey’s multiple comparison tests was performed. For non−normally distributed data, or when the sample size was too small to determine normality, nonparametric analysis was performed: Mann−Whitney U test (two−tailed) for two groups, or Kruskal-Wallis test with post−hoc Dunn’s tests for three or more groups. Adjusted *p* values were used, and *p* < 0.05 was considered statistically significant. All data were analyzed using GraphPad Prism 10.0 software.

## 3 Results

### 3.1 miR-1a-3p could directly target *Fcgr4*

**Figure 1:**
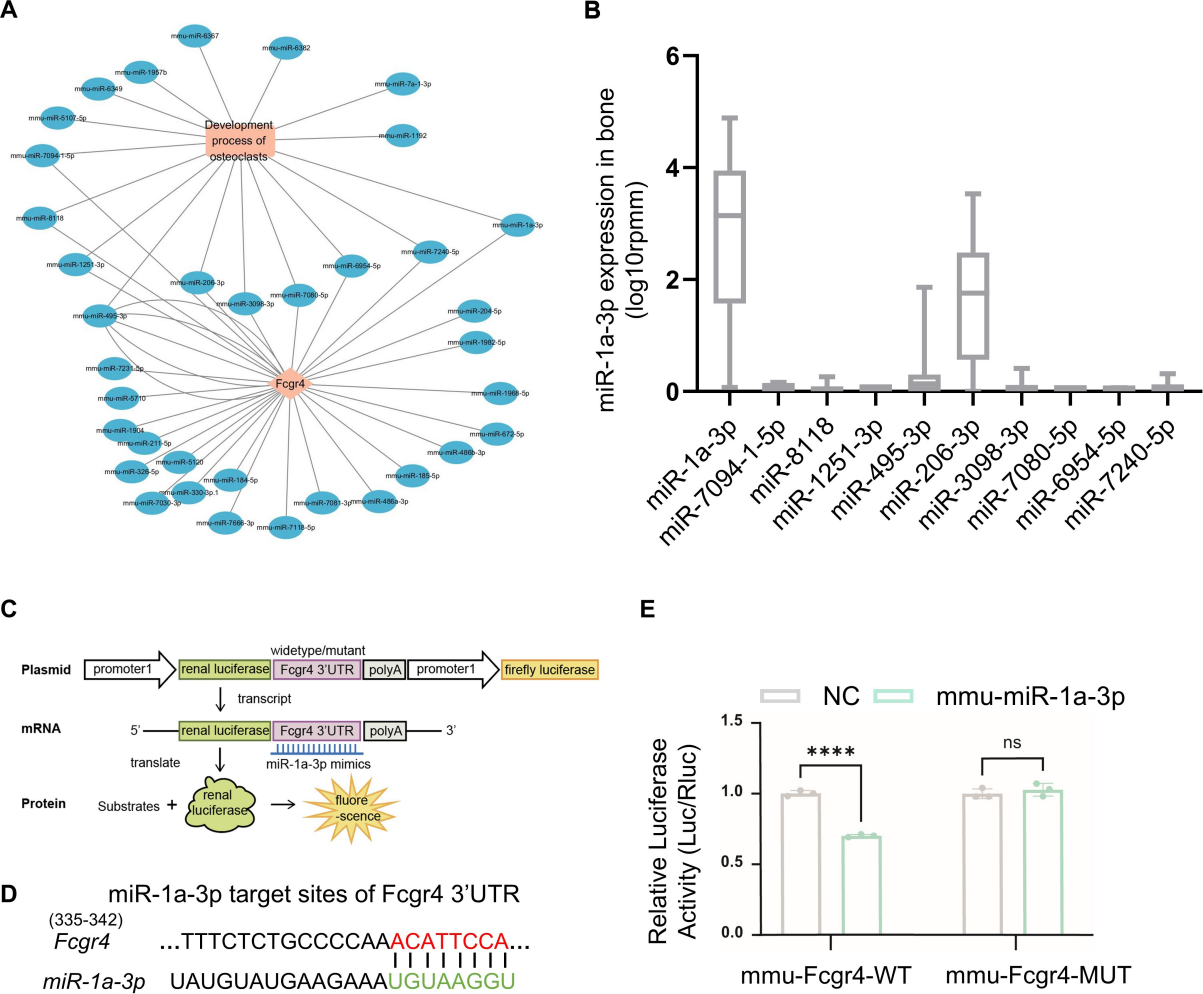
Identify and verify microRNAs targeting *Fcgr4*. **(A)** Analysis of *Fcgr4* and its predictive target microRNAs. **(B)** Expression of different types of microRNAs in Bone (data from the miRNA Tissue Atlas 2025: https://web.ccb.uni-saarland.de/mirnatissueatlas_2025). **(C)** Construction of a luciferase reporter gene plasmid. **(D)** Sequence alignment of miR-1a-3p and its predicted target sites in 3′UTR of *Fcgr4*. **(E)** Luciferase activity (n=3). All data are presented as mean ± SD; ns, *p*>0.05, ****p < 0.0001.

Firstly, to identify candidate miRNAs that may regulate *Fcgr4*, *Fcgr4*-targeting miRNAs associated with osteoclast differentiation and maturation were screened using DIANA and TargetScan (**Figure 1A**). Among the predicted candidates, miR-1a-3p exhibited relatively high expression in bone, suggesting a potential role in the regulating bone metabolism (**Figure 1B**). Then, to determine whether miR-1a-3p directly targets *Fcgr4*, a luciferase reporter plasmid containing the predicted miR-1a-3p binding site was constructed (**Figure 1C**). Co-transfection with miR-1a-3p significantly reduced the luciferase activity of the wild-type reporter, whereas no appreciable change was observed in the mutant reporter lacking the predicted binding site (**Figure 1E**). Collectively, these results confirmed that miR-1a-3p directly targets *Fcgr4*.

### 3.2 miR-1a-3p could restrain *Fcgr4*-associated osteoclast activity

**Figure 2:**
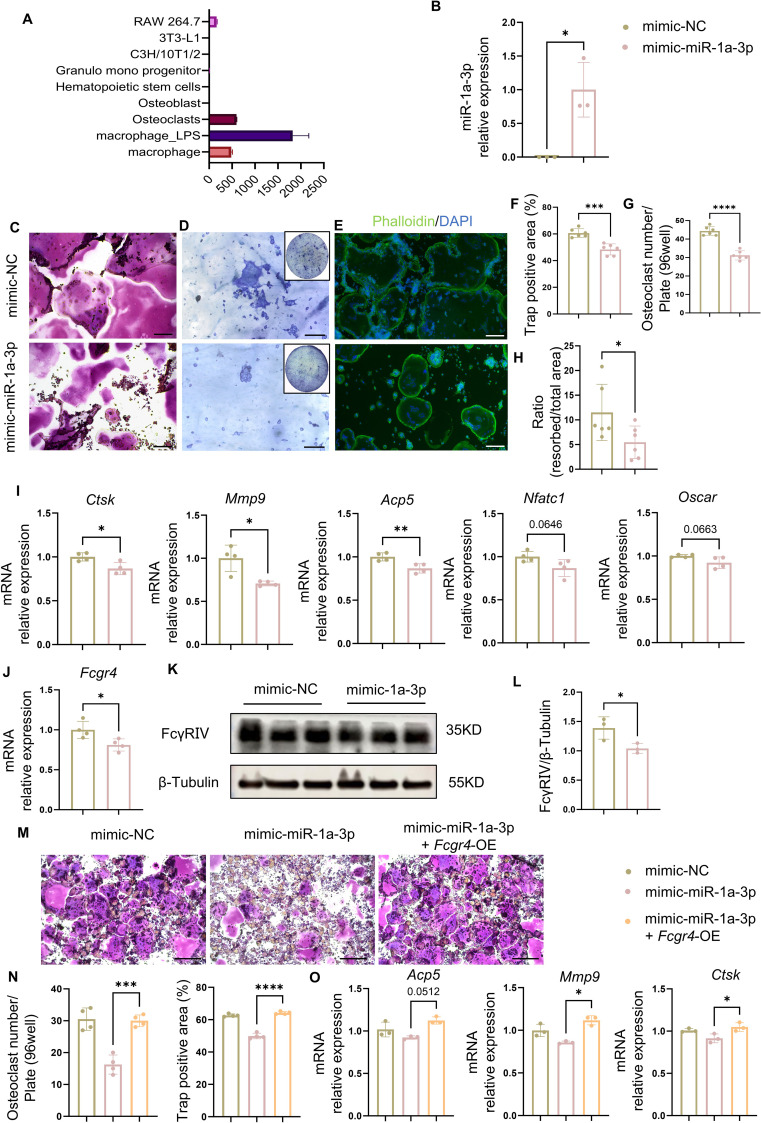
The role of miR-1a-3p through Fcgr4 in osteoclast activity. **(A)** Expression of *Fcgr4* in different cells (data from http://biogps.org/#goto=genereport&id=246256). **(B)** Transfection efficiency (n=3). **(C)** Representative TRAP staining images. Scale bar, 200µm. **(D)** Representative resorption pits. Scale bar, 200µm. **(E)** Representative phalloidin staining images. Scale bar, 200µm. **(F)** The TRAP positive area of osteoclasts (n=6). **(G)** The amounts of osteoclasts (multinucleated TRAP-positive cells) (n=6). **(H)** The area of resorbed surface (n=6). **(I)** qRT-PCR quantification analysis of *Ctsk*, *Mmp9, Acp5, Nfatc1, Oscar* expression of RANKL-induced RAW264.7 transfected with mimic-NC and mimic-miR-1a-3p (n=4). **(J)** qRT-PCR quantification analysis of *Fcgr4* expression of RANKL-induced RAW264.7 transfected with mimic-NC and mimic-miR-1a-3p (n=4). **(K)** The protein expression of FcγRIV. **(L)** Semi-quantification analysis of FcγRIV (n=3). **(M)** Representative TRAP staining images. Scale bar, 500µm. **(N)** Quantitative analysis of the amounts of osteoclasts and positive area of osteoclasts (n=4). **(O)** qRT-PCR quantification analysis of *Acp5, Ctsk*, *Mmp9* expression of RANKL-induced RAW264.7 transfected with mimic-NC, mimic-miR-1a-3p and mimic-miR-1a-3p+*Fcgr4*-OE (n=3). All data are presented as mean ± SD; *p < 0.05, **p < 0.01, ***p < 0.001, ****p < 0.0001.

Given that FcγRIV is highly expressed in macrophages and osteoclasts (**Figure 2A**) and is involved in osteoclast activation (14–17), we next examined whether miR-1a-3p affects osteoclastogenesis through *Fcgr4*. As shown in **Figure 2B**, transfection with mimic-miR-1a-3p significantly increased miR-1a-3p expression. After RANKL stimulation, TRAP staining, the bone resorption pit assays and phalloidin staining revealed impaired the activity and function of osteoclasts in mimic-miR-1a-3p group (**Figures 2C–H**). Consistently, the expression of osteoclast-related genes, including *Ctsk*, matrix metallopeptidase 9 (*Mmp9*), *Acp5, Nfatc1*, and osteoclast associated Ig-like receptor (*Oscar)*, was also reduced after miR-1a-3p overexpression (**Figure 2I**). Notably, *Fcgr4* expression was decreased at both mRNA and protein levels following miR-1a-3p overexpression (**Figures 2J–L**). To further assess whether *Fcgr4* mediated the observed suppression, *Fcgr4* was rescued in miR-1a-3p-overexpressing cells. TRAP staining (**Figures 2M,N**) and qRT-PCR (**Figure 2O**) showed that Fcgr4 restoration could reverse the miR-1a-3p induced suppression of osteoclast formation and related gene expression. Together, these findings indicated that miR-1a-3p negatively regulates osteoclastic activity through suppression of *Fcgr4* expression.

### 3.3 miR-1a-3p significantly decreased across pathological osteoporosis

**Figure 3:**
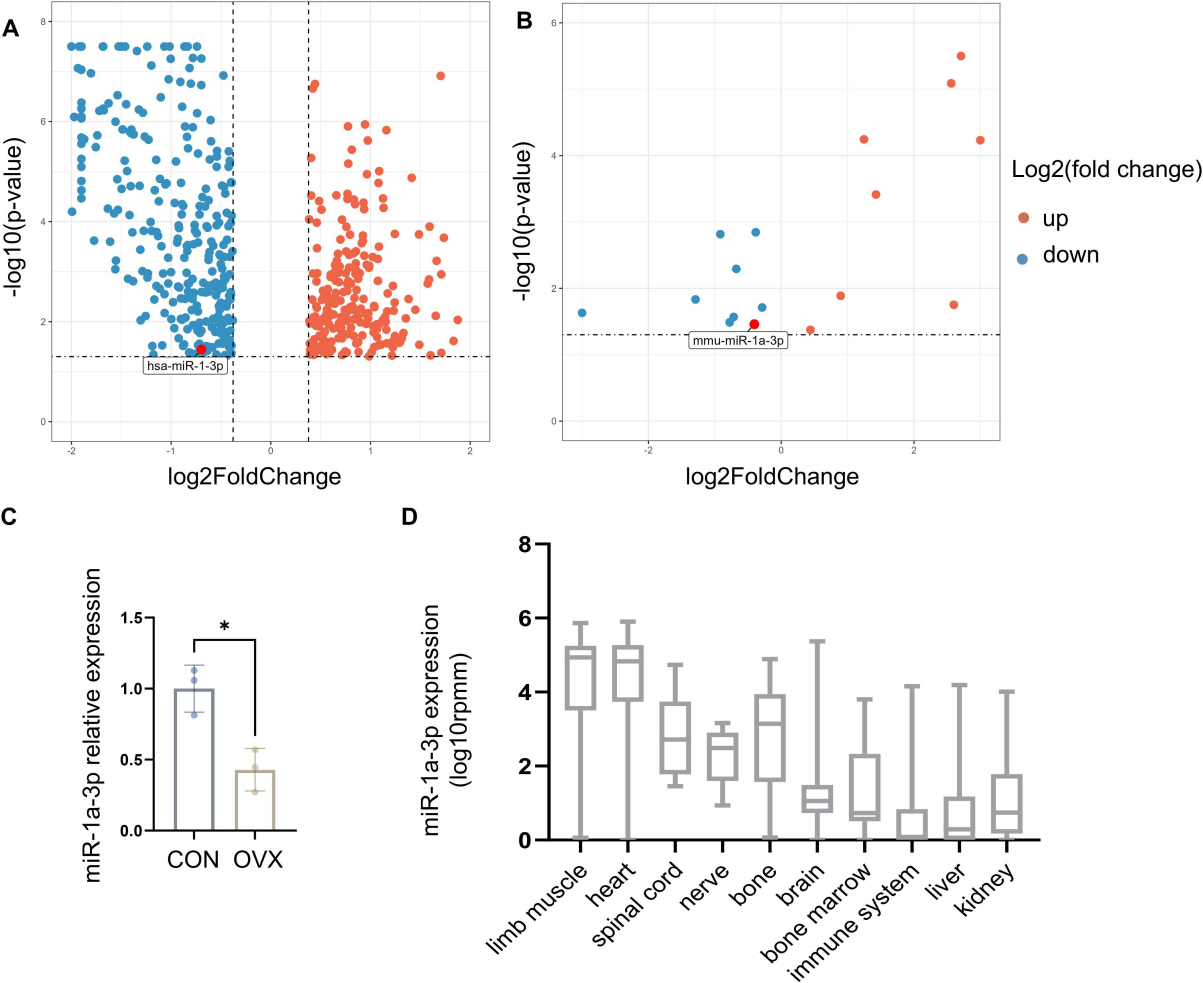
Alterations in miR-1a-3p in pathological osteoporosis. **(A)** Volcano plot for visualizing of differentially expressed miRNAs in human osteoporosis cohorts. **(B)** Volcano plot for visualizing of differentially expressed miRNAs in OVX mice. **(C)** Analysis of miR-1a-3p relative expression levels. **(D)** Expression of miR-1a-3p in different organs (data from the miRNA Tissue Atlas 2025: https://web.ccb.uni-saarland.de/mirnatissueatlas_2025). All data are presented as means ± SD. ns, *p >*0.05, *p < 0.05, vs. the CON group.

Then, to assess the involvement of miR-1a-3p in pathological bone loss, we examined its expression in public miRNA profiling datasets from human osteoporosis cohorts and established animal models of osteoporosis. In human osteoporosis cohorts, miR-1a-3p expression was significantly lower than that in healthy control (**Figure 3A**; **Supplementary Table 1**). A similar decrease was detected in the femoral trabecular bone of ovariectomized (OVX) mice compared with sham-operated controls (**Figures 3B,C**; **Supplementary Table 1**). In addition, tissue distribution analysis revealed that miR-1a-3p was not preferentially enriched in bone and exhibits high basal expression in neural and muscle tissues (**Figure 3D**). These findings show that miR-1a-3p is reduced in osteoporosis across independent human and mouse datasets and is not preferentially enriched in bone, supporting the possibility that its dysregulation may occur across distinct systemic pathological contexts associated with bone loss.

### 3.4 Psychological stress is associated with reduced miR-1a-3p expression, enhanced Fcgr4-related osteoclast activity, and bone loss

**Figure 4:**
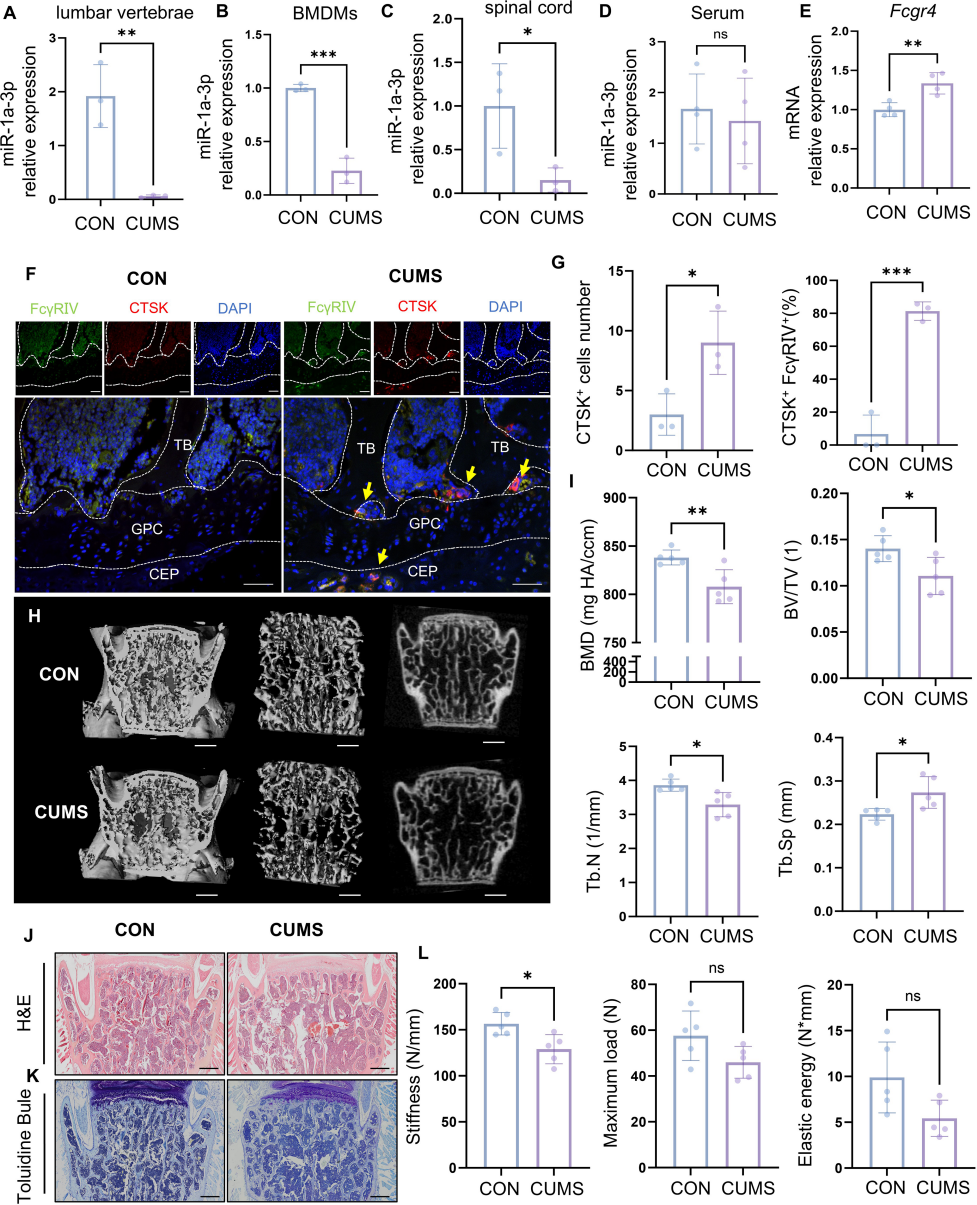
Psychological stress is associated with miR-1a-3p downregulation and osteoclast-driven lumbar vertebral bone loss. **(A)** qRT-PCR quantification analysis of miR-1a-3p in lumbar vertebrae (n=3). **(B)** qRT-PCR quantification analysis of miR-1a-3p in BMDMs (n=3). **(C)** qRT-PCR quantification analysis of miR-1a-3p in spinal cord (n=3). **(D)** Serum miR-1a-3p levels (n=4). **(E)** qRT-PCR quantification analysis of *Fcgr4* in BMDMs (n=4). **(F)** Representative IF images for FcγRIV, CTSK, and DAPI expression. Scale bar, 50µm. **(G)** Quantitative analysis of CTSK^+^ and FcγRIV^+^ cells. **(H)** Representative X-ray images of lumbar spine from the CON and the CUMS mice. Scale bar, 1mm. **(I)** Quantitative micro-CT analysis of lumbar vertebrae (n=5). **(J)** H&E staining. Scale bar, 500µm ***p<0.001. **(K)** Toluidine Blue staining. Scale bar, 500µm. **(L)** Results of compression test (n=5). All data are presented as means ± SD. ns, *p >*0.05, *p < 0.05, **p < 0.01 vs. the CON group. CEP, cartilaginous endplate; GPC, growth plate cartilage; TB, trabecular bone.

To further assess whether miR-1a-3p responds to a defined systemic stress condition, we examined its expression and changes related to bone remodeling in the CUMS model, as miR-1a-3p has previously been reported to be a psychological stress-responsive miRNA (28–30). Following 6 weeks of CUMS exposure, mice exhibited robust depressive- and anxiety-like behaviors, confirming successful establishment of the CUMS model (**Supplementary Figure 1**). miR-1a-3p expression was significantly reduced in the lumbar vertebrae (**Figure 4A**), BMDM cells (**Figure 4B**), and the spinal cord (**Figure 4C**) of CUMS mice compared with CON mice, whereas circulating miR-1a-3p levels in serum remained unchanged (**Figure 4D**). In parallel, *Fcgr4* mRNA expression in BMDMs (**Figure 4E**) and FcγIV protein level in the lumbar vertebrae (**Supplementary Figure 2A**) were elevated in CUMS mice relative to CON mice.

Immunofluorescence analysis further revealed increased colocalization of FcγRIV with the osteoclast marker CTSK in lumbar vertebrae of CUMS mice (**Figure 4F**). Quantitative analysis showed an increase in the number of CTSK-positive cells, and a higher proportion of FCγRIV-positive among CTSK-positive cells in CUMS group (**Figure 4G**). Consistently, RT-qPCR, IHC and TRAP staining of lumbar vertebrae, together with *in vitro* osteoclast induction of BMDMs, indicated increased osteoclast activity and function under psychological stress (**Supplementary Figure 2**). Besides, osteogenic activity was also enhanced, measuring by calcein double labeling, IHC for OSX, OCN, and OPN, and qRT-PCR of osteoblast-related gene, including osteocalcin (*Ocn)*, osteopontin (*Opn)*, alkaline phosphatase (*Alp)*, runt-related transcription factor 2(*Runx2)* and dentin matrix protein 1(*Dmp1)* (**Supplementary Figure 3**).

Nevertheless, micro-CT showed a significant reduction in trabecular bone mass in the lumbar spine of CUMS mice (**Figures 4H, I**), with similar changes in the femur and tibia (**Supplementary Figures 4A–D**). Consistently, H&E staining (**Figure 4J**) and toluidine blue staining (**Figure 4K**) showed loss of trabecular structure in the lumbar vertebrae of CUMS mice. In line with these structural changes, mechanical testing demonstrated reduced lumbar vertebrae stiffness in CUMS mice, whereas maximum load and elastic energy were not significantly changed (**Figure 4L**). Taken together, this evidence all implied that chronic psychological stress is associated with increased Fcgr4-related osteoclastic activity and reduced bone mass, accompanied by decreased miR-1a-3p expression.

### 3.5 Activation of the FcγIV–SYK–NFATc1 signaling axis in osteoclasts under psychological stress

**Figure 5:**
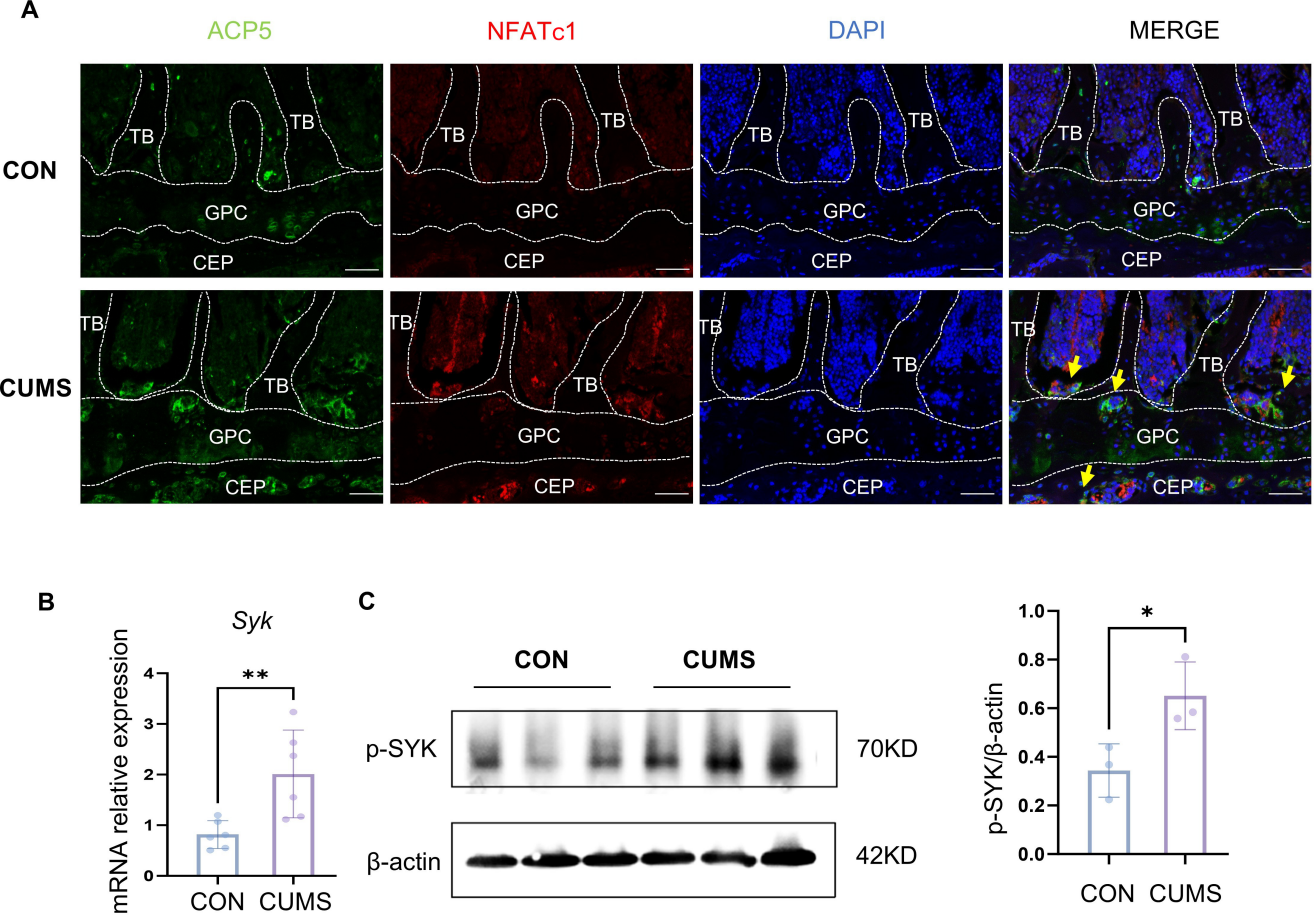
miR-1a-3p downregulation is associated with activation of osteoimmune pathway in lumbar vertebral osteoclasts. **(A)** Representative IF images for ACP5, NFATc1, and DAPI expression. Scale bar, 50µm. **(B)** qRT-PCR quantification analysis of *Syk* (n=6). **(C)** The protein expression and quantification of p-SYK (n=3). All data are presented as mean ± SD, ns, *p*>0.05, *p < 0.05, **p < 0.01 vs. the CON group. CEP, cartilaginous endplate; GPC, growth plate cartilage; TB, trabecular bone.

Next, to elucidate the mechanisms underlying the above response to psychological stress, the key downstream pathways of FcγIV were subsequently examined. Immunofluorescence analysis revealed increased colocalization of ACP5 with NFATc1 in the lumbar spine of CUMS mice (**Figure 5A**). In parallel, both SYK and phosphorylated SYK (p-SYK) levels, key downstream effectors of FcγRIV (31), were significantly elevated in lumbar vertebrae from CUMS mice (**Figures 5B, C**). These findings showed activation of the FcγIV–SYK–NFATc1 signaling axis in osteoclasts under chronic psychological stress, and supported a model in which psychological stress-associated downregulation of miR-1a-3p is accompanied by enhanced Fcgr4-dependent signaling, promoting osteoclast activation and bone loss.

## 4 Discussion

**Figure 6:**
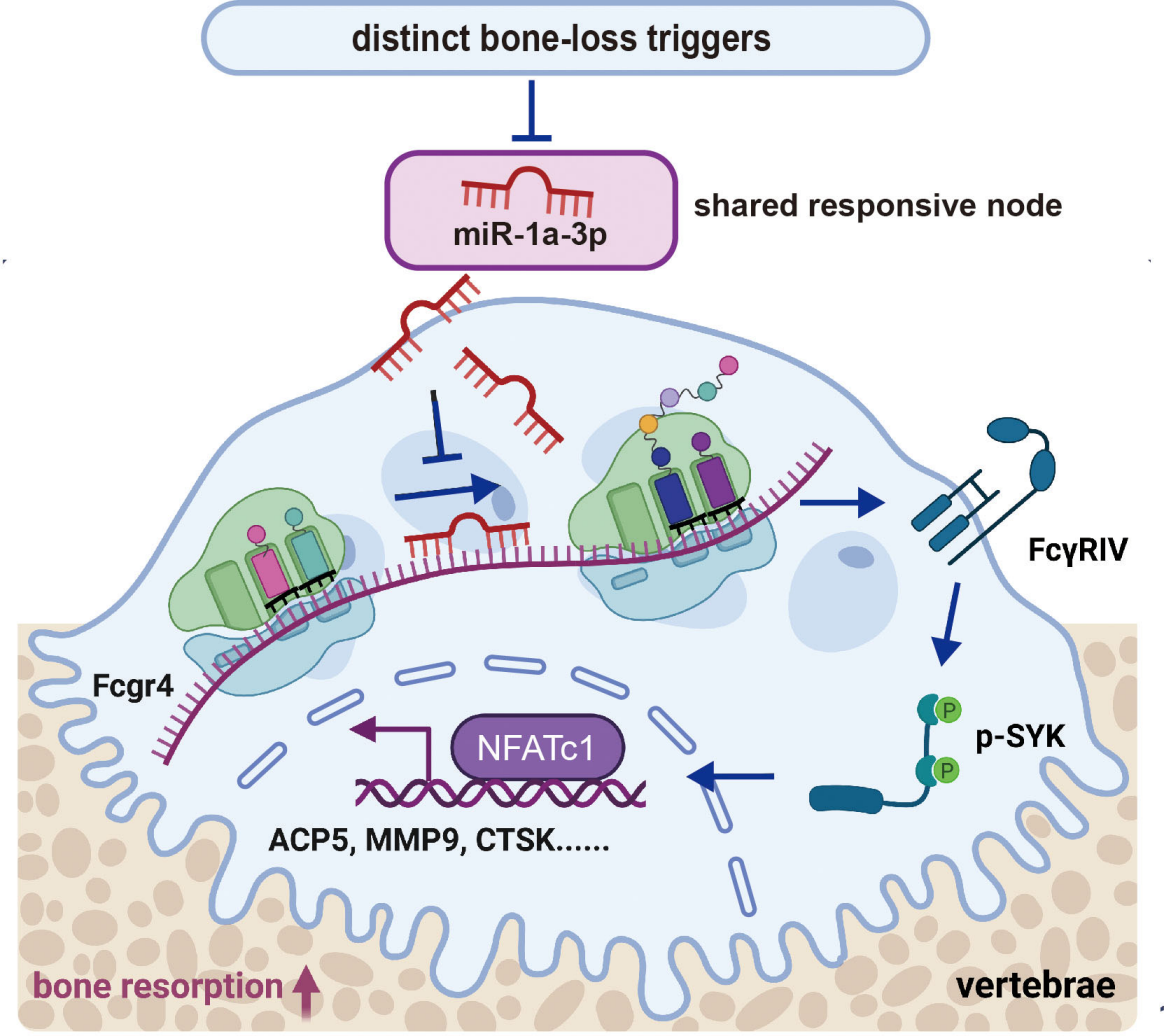
Proposed schematic diagram of the miR-1a-3p/*Fcgr4* axis in in pathological bone-loss (Created in https://BioRender.com).

Immunoreceptor-dependent osteoclast activation may represents an important route through which non–bone-intrinsic signals drive pathological bone resorption. Here, we show that miR-1a-3p expression is reduced in models of bone loss induced by estrogen deficiency and psychological stress. This reduction could relieve post-transcriptional repression of its target gene, *Fcgr4*, thereby increase FcγRIV availability, and promote osteoclast activation (**Figure 6**). Taken together, our findings support the miR-1a-3p/*Fcgr4* axis as a potential shared downstream regulatory node through which distinct systemic pathological conditions converge on osteoclast dysregulation and bone loss, which may provide a potential entry point for future therapeutic investigation.

miRNAs represent a regulatory layer well positioned to link systemic perturbations to bone remodeling (32). Unlike classical inflammatory mediators, which often reflect acute immune activation, miRNAs are relatively stable in body fluids (33–35) and can remain dysregulated across disease progression, thereby enabling more durable regulatory states. Through post-transcriptional modulating target mRNAs, miRNA could adjust signaling thresholds and cellular responsiveness, making them especially pertinent to conditions such as psychological stress and estrogen deficiency, in which neural, endocrine, and immune inputs are persistently altered rather than acutely activated within bone tissue. In this context, the incomplete protection of bone mass by interventions targeting established stress-related pathways (36–38), such as antidepressant treatment (39) or β-adrenergic blockade (40), further suggests that stress-associated bone loss cannot be fully explained by these pathways alone. miRNA-mediated regulation may therefore represent an additional mechanism contributing to sustained osteoclast activation. This regulatory property also gives miRNAs translational relevance, not only as candidate biomarkers of sustained systemic dysregulation but also as potential intervention points. Indeed, accumulating preclinical studies in osteoporosis have demonstrated that modulation of specific miRNAs can attenuate bone-loss phenotypes (41, 42). Together, these suggest that miRNAs may serve as actionable regulatory targets in pathological bone loss.

Under physiological conditions, miR-1a-3p is highly enriched in excitable tissues, particularly cardiac and skeletal muscle, under physiological conditions (43, 44). Most previous studies have focused on its tissue-intrinsic roles. In those contexts, reduced miR-1a-3p expression has been reported to protect cardiomyocytes and improve cardiac function in a mouse model of heart failure, while also promoting skeletal muscle growth through activation of the Akt/mTOR/S6K pathways (45). Together, these findings establish miR-1a-3p as a regulator of cellular growth and stress responses within its tissues of origin. However, its role beyond organ-autonomous regulation has remained insufficiently defined, particularly in immune-related and bone-associated pathological settings. In the present study, our data support miR-1a-3p as a previously unrecognized regulator of the osteo–immune axis that modulates osteoclast activation through post-transcriptional control of the immunoreceptor Fcgr4, thereby extending its functional relevance beyond muscle and cardiac tissues to pathological bone remodeling.

Our study showed that miR-1a-3p expression is reduced in osteoporosis patients, OVX mice, and CUMS mice, suggesting that this miRNA may act as a shared downstream responder across distinct etiological forms of osteoporosis. Notably, in the CUMS model, reduced miR-1a-3p expression in bone was not accompanied by a detectable change in serum. This finding suggests that miR-1a-3p regulation is more likely to occur in a tissue-selective manner within the skeletal microenvironment, rather than a circulating endocrine-like mechanism. This is consistent with previous observations that, in bone-related disorders, miRNA alterations across serum, bone marrow, and bone often show only partial overlap and may even differ in direction (46). Together, these observations further suggest that systemic pathological inputs may regulate the expression of miR-1a-3p in bone, potentially through neural, neuroendocrine, or other locally acting factors.

Therefore, the upstream mechanisms responsible for miR-1a-3p reduction are likely distinct across these models. In CUMS model, systemic neuroendocrine remodeling, particularly the activation of the sympathetic-adrenal medulla axis may be a key driver (47). Accordingly, β-adrenergic receptor signaling is activated and acts through the cAMP/PKA pathway, which has been implicated in both bone loss (48) and miRNA reprogramming (49). Because PKA-dependent HDAC4 signaling can suppress MEF2 (50), a known transcriptional activator of miR-1a-3p (51), which provides a potential mechanism by which chronic stress downregulate miR-1a-3p.In contrast, the decrease in miR-1a-3p seen in OVX is more likely to be explained by reduced estrogen levels (25). Estrogen signaling has been shown to enhance SRE/SRF-dependent transcription, suggesting that estrogen withdrawal may weaken SRF-mediated miR-1a-3p transcription. In addition, estrogen deficiency may promote DNMT1/3a/3b upregulation (52) and HDAC-mediated chromatin repression in bone (53, 54), both of which could contribute to miR-1a-3p silencing (55, 56). However, despite distinct upstream triggers, CUMS and OVX may converge on miR-1a-3p, underscoring its central role in responding to etiologically distinct systemic inputs to osteoclast dysregulation and bone loss.

Osteoclasts were the primary focus of this study because their myeloid origin, immunoreceptor repertoire, and bone-resorptive function make them central to osteoimmune dysregulation. Nevertheless, because bone mass result from the balance between resorption and formation, osteoblast-related parameters were also assessed to characterize the overall remodeling status. In the CUMS model established using 7-week-old mice, bone formation was found to be slightly increased, which is consistent with previous observations in mice of a similar age (57) but differs from reports in older mice (48). This discrepancy may be indicative of a compensatory response that is dependent on age or stage (58) during the process of stress-induced bone remodeling. Importantly, trabecular bone mass was ultimately reduced, suggesting that this osteogenic response was insufficient to offset excessive resorption. Thus, osteoclast activation remains the major contributor to CUMS-induced bone loss and the central mechanistic focus of this study.

Although we have demonstrated that decreased miR-1a-3p significantly increases *Fcgr4* expression and promotes osteoclast activation, the upstream regulators responsible for miR-1a-3p downregulation in osteoclast-lineage cells under estrogen deficiency or chronic stress were not directly examined in this study. Particularly, stress-related hormones and neurotransmitters, such as corticosterone and catecholamines, were not measured in the CUMS model. Therefore, the present data cannot determine to what extent glucocorticoid- or sympathetic-mediated effects contribute directly to the observed bone loss or act upstream of the miR-1a-3p/Fcgr4 axis. In addition, whether additional regulatory layers, such as metabolic reprogramming or epigenetic regulation, act in parallel with miR-1a-3p–mediated post-transcriptional regulation remains unclear. Moreover, given that miRNAs can target multiple genes (59), miR-1a-3p may also affect osteoclast function through other immune- or metabolism-related genes other than *Fcgr4*, which requires further study. Furthermore, while our experiments *in vitro* have showed that miR-1a-3p overexpression suppresses osteoclastogenesis, its direct *in vivo* contribution to bone loss phenotypes remains to be established. In particular, whether restoring miR-1a-3p can attenuate bone loss in CUMS or OVX models requires further investigation.

In conclusion, this study supports a miRNA-dependent mechanism in which reduced miR-1a-3p expression contributes to sustained osteoclast activation through increased availability of the immunoreceptor Fcgr4, thereby adding a new regulatory layer to current models of chronic bone loss.

## Data Availability

The raw data supporting the conclusions of this article will be made available by the authors, without undue reservation.
