# Supplementary material for: MiR-1a-3p/Fcgr4-dependent osteoclast activation regulates pathological bone loss
Source: Front Immunol. 2026 May 25;17:1828877. doi: 10.3389/fimmu.2026.1828877 (PMC13243279; doi:10.3389/fimmu.2026.1828877)
Supplement: Supplementary file 1 [file Supplementaryfile1.docx]

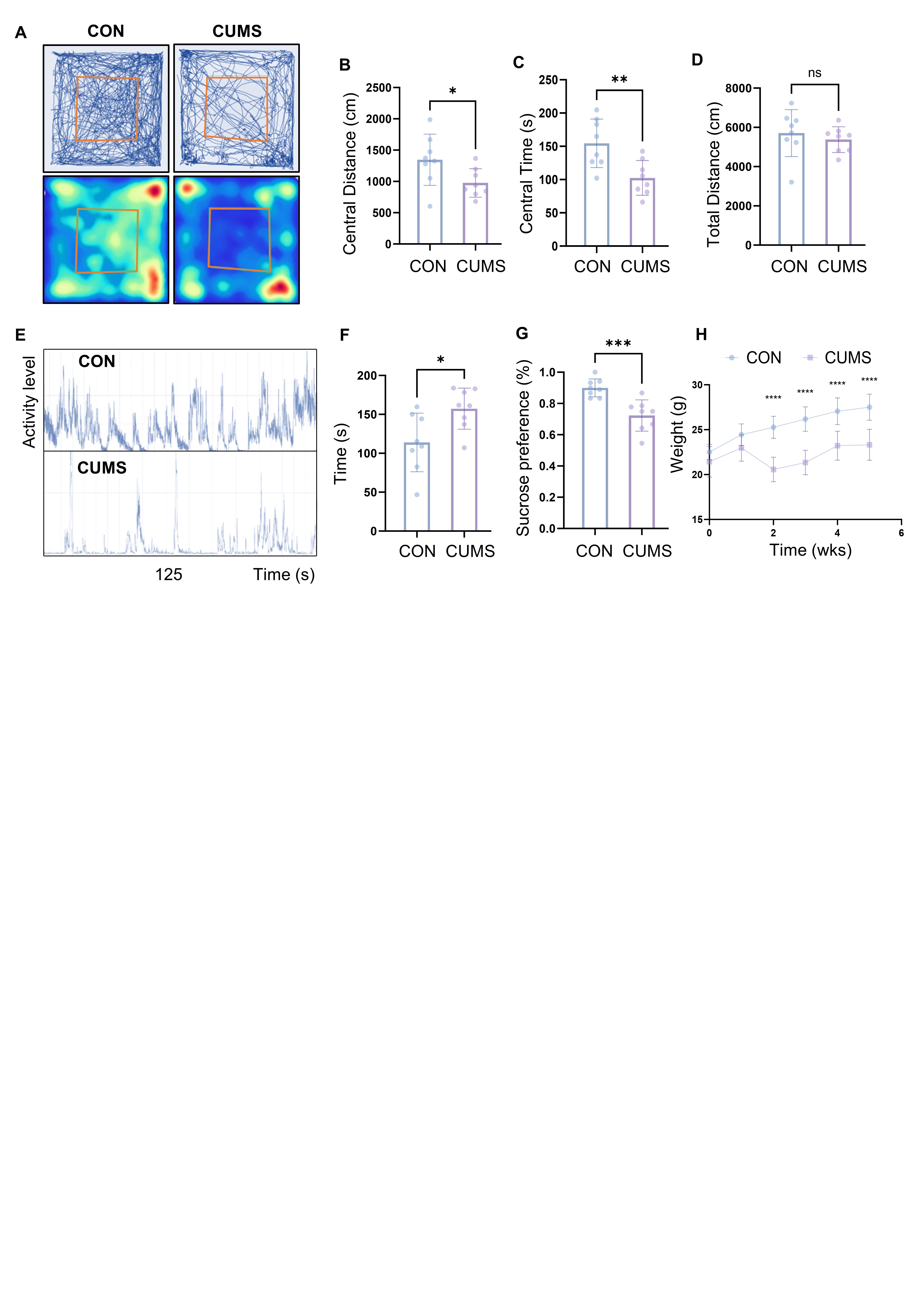


**Supplement Figure1** Effects of the CUMS model on behavioral performance and body weight of mice. (A) Representative movement trajectories of mice in the OFT. Distance (B) and time of movement (C) in the central area in the OFT. (D) Total distance moved in the OFT. (E) Representative waveforms of mouse activity levels in the TST. (F) Immobility time of mice in the TST. (G) Sucrose preference index. (H) Body weight. All data are presented as means ± SD (n=8). *, P < 0.05, **, P<0.01, ***, P < 0.001, ****, P<0.0001 vs. the CON group.


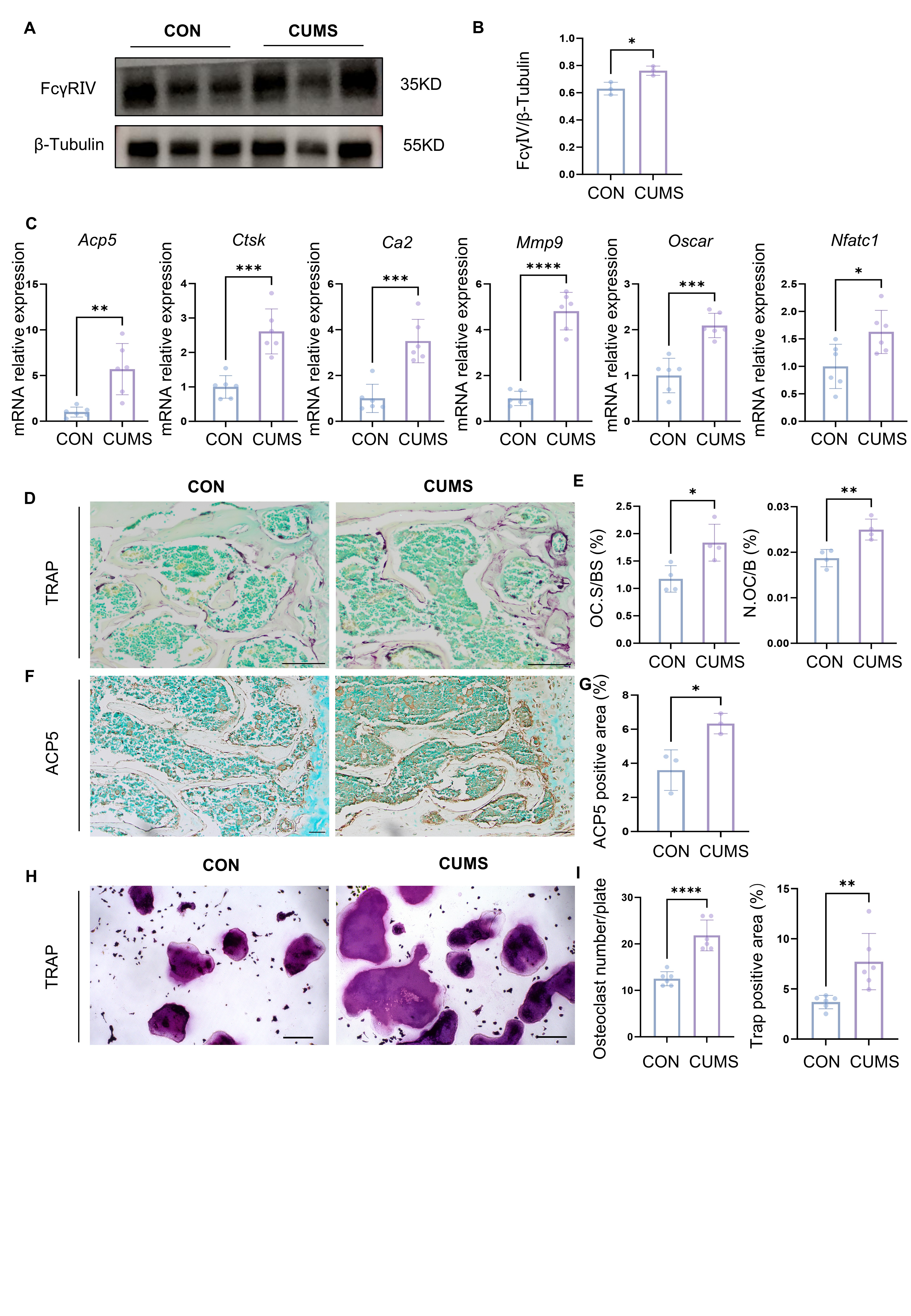


**Supplement Figure 2** Changes in osteoclast activity under psychological stress. (A) The protein expression of FcγRⅣ in lumbar vertebral. (B) Semi-quantification analysis of FcγRⅣ (n=3). (C) qRT-PCR quantification analysis of osteoclastic markers including*Acp5*, *Ctsk*, *Ca2*, *Mmp9*, *Oscar*, *Nfatc1* (n=6). (D-E) Representative TRAP staining images of lumbar vertebrae with quantitative analysis of osteoclast surface per bone surface (Oc. S/BS) and number per bone surface (N. OC/B) (n=4). Scale bar, 100µm. (F) Representative IHC images for ACP5 expression. Scale bar, 100µm. (G) Quantitative analysis of ACP5 in the lumbar spine (n=3). (H) TRAP staining of RNAKL-induced BMDMs. Scale bar, 200µm. (I) Quantitative analysis of the amounts of osteoclasts and positive area of osteoclasts (n=6). All data are presented as means ± SD. *, *p* < 0.05, **, *p* <0.01, ***, *p* < 0.001, ****, *p* <0.0001 vs. the CON group.


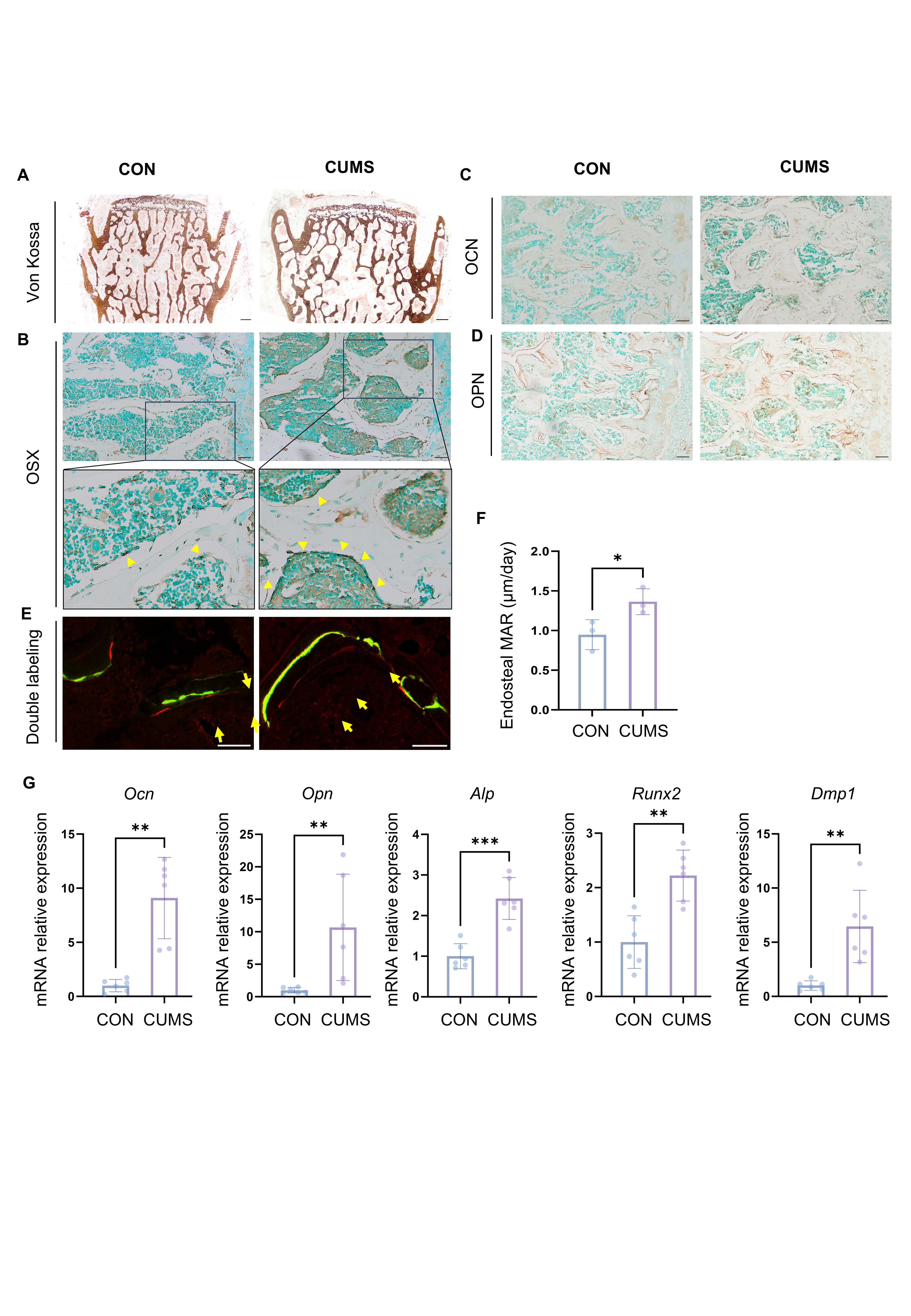


**Supplement Figure 3** Osteogenic metabolism of lumbar vertebrae under psychological stress. (A) Von kossa staining. Scale bar, 200µm (B-D) Representative IHC images for OSX, OCN, OPN expression. Scale bar, 100µm. (E) Fluorescent double labeling of lumbar vertebrae. Scale bar, 50um. (F) Quantification analysis of MAR (n=3). (G) qRT-PCR quantification analysis of osteogenic markers including *Ocn*, *Opn*, *Alp*, *Runx2*, *Dmp1* (n=6). All data are presented as mean ± SD, ns, *p* >0.05, *, *p* < 0.05, **, *p* <0.01, ***, *p* < 0.001 vs. the CON group.


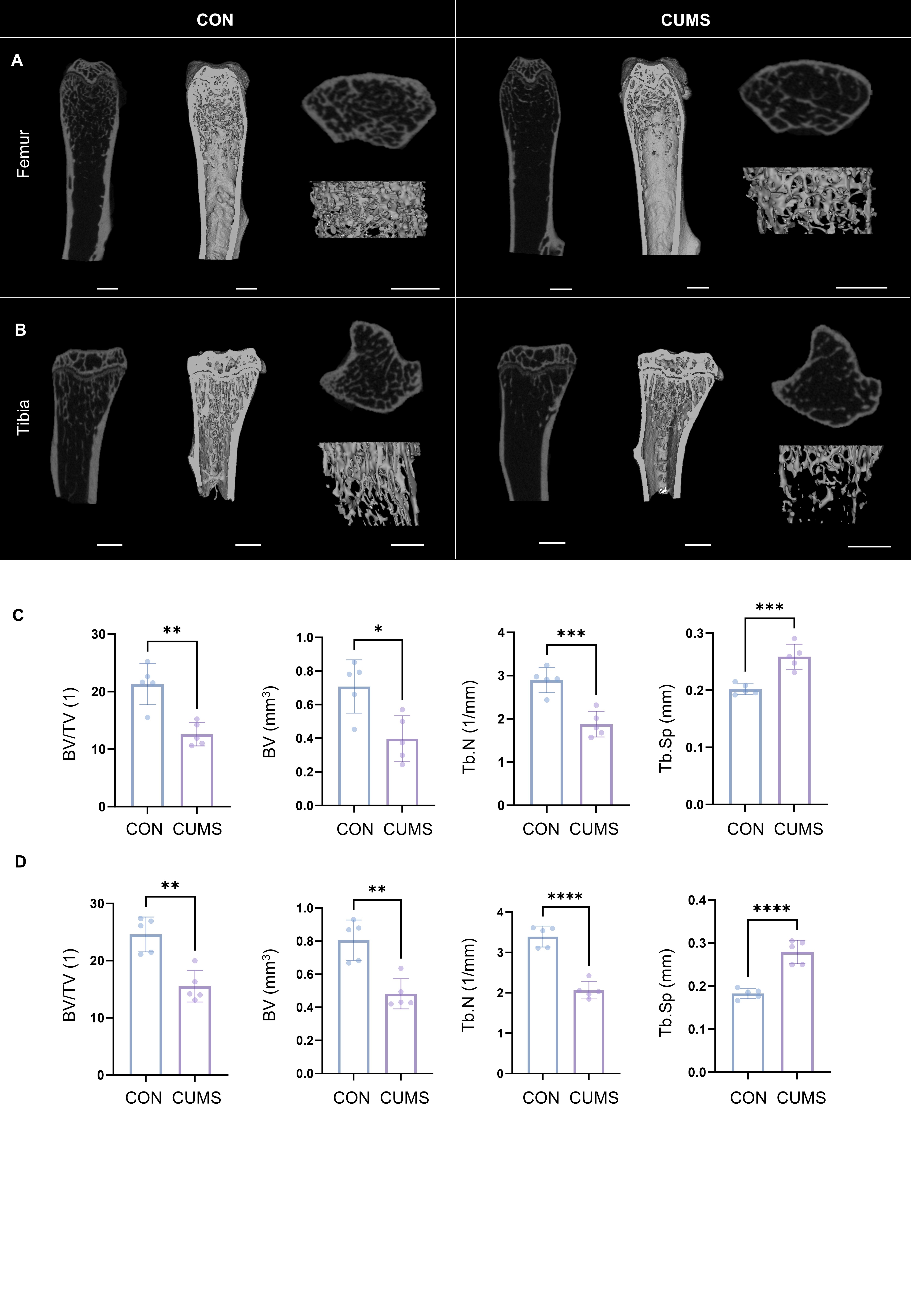


**Supplement Figure 4** Changes in the trabecular bone of the femur and tibia under psychological stress. (A) Representative X-ray images of femurs from control and CUMS mice. (B) Representative X-ray images of tibias from control and CUMS mice. (C) Quantitative micro-CT analysis of femur trabecular bone. (D) Quantitative micro-CT analysis of tibia trabecular bone. Scale bar, 100µm. All data are presented as means ± SD (n=5). *, *p* < 0.05, **, *p* <0.01, ***, *p* < 0.001, ****, *p* <0.0001 vs. the CON group.

**Supplement Figure 5** Full unedited gel for Western Blot.

In Fig.2K：


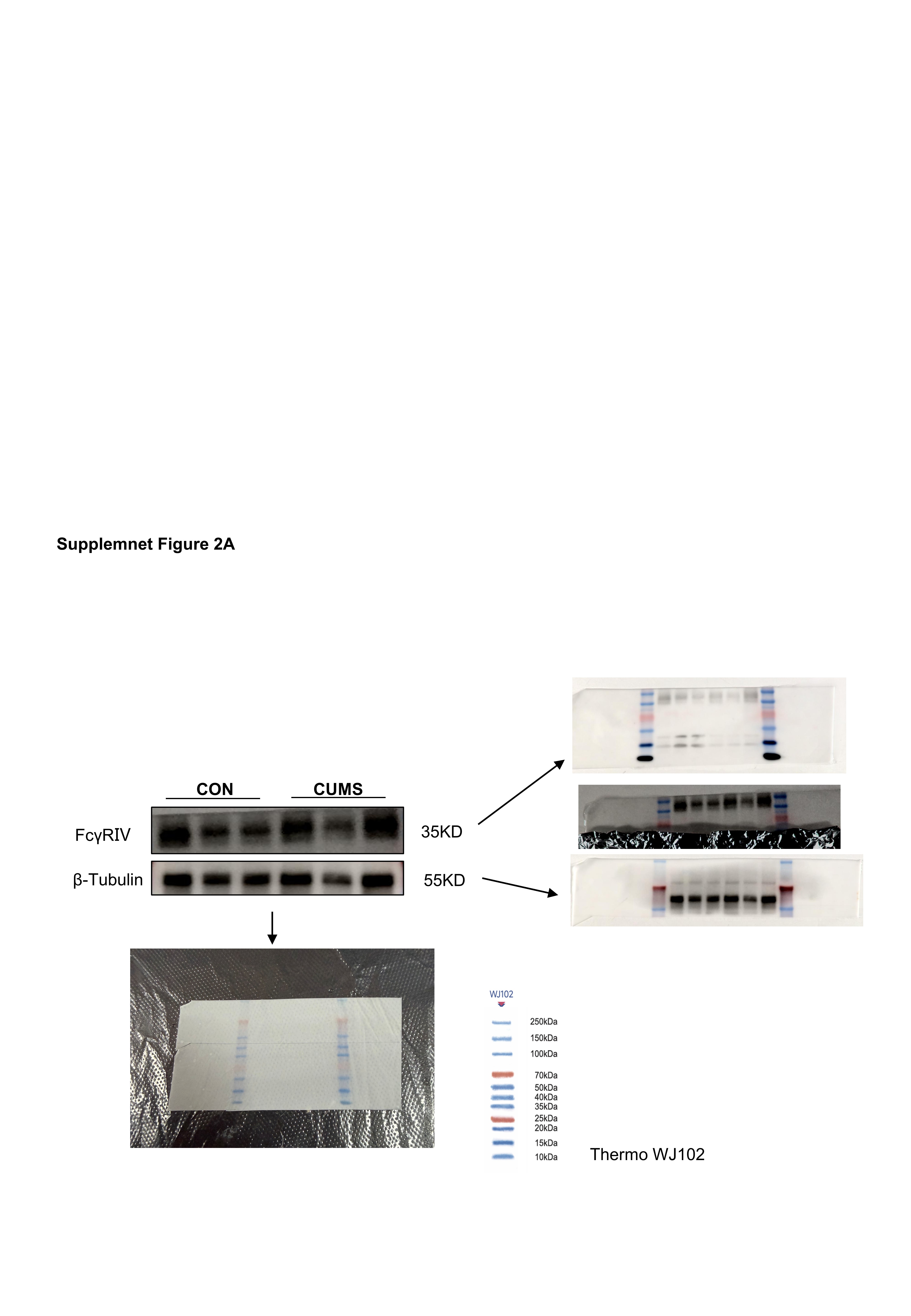


In Fig. 5C：


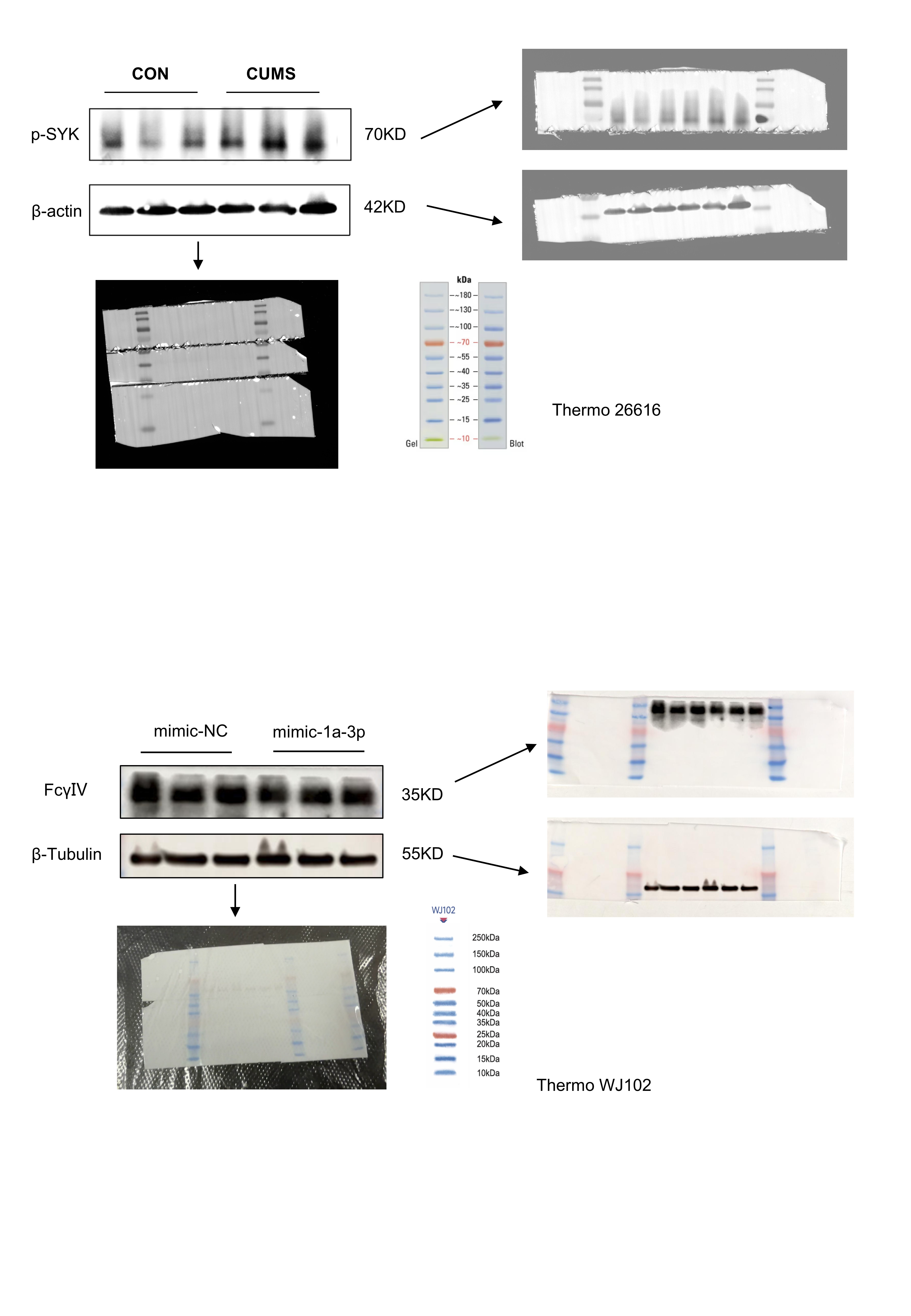


In Supplement fig. 2A:


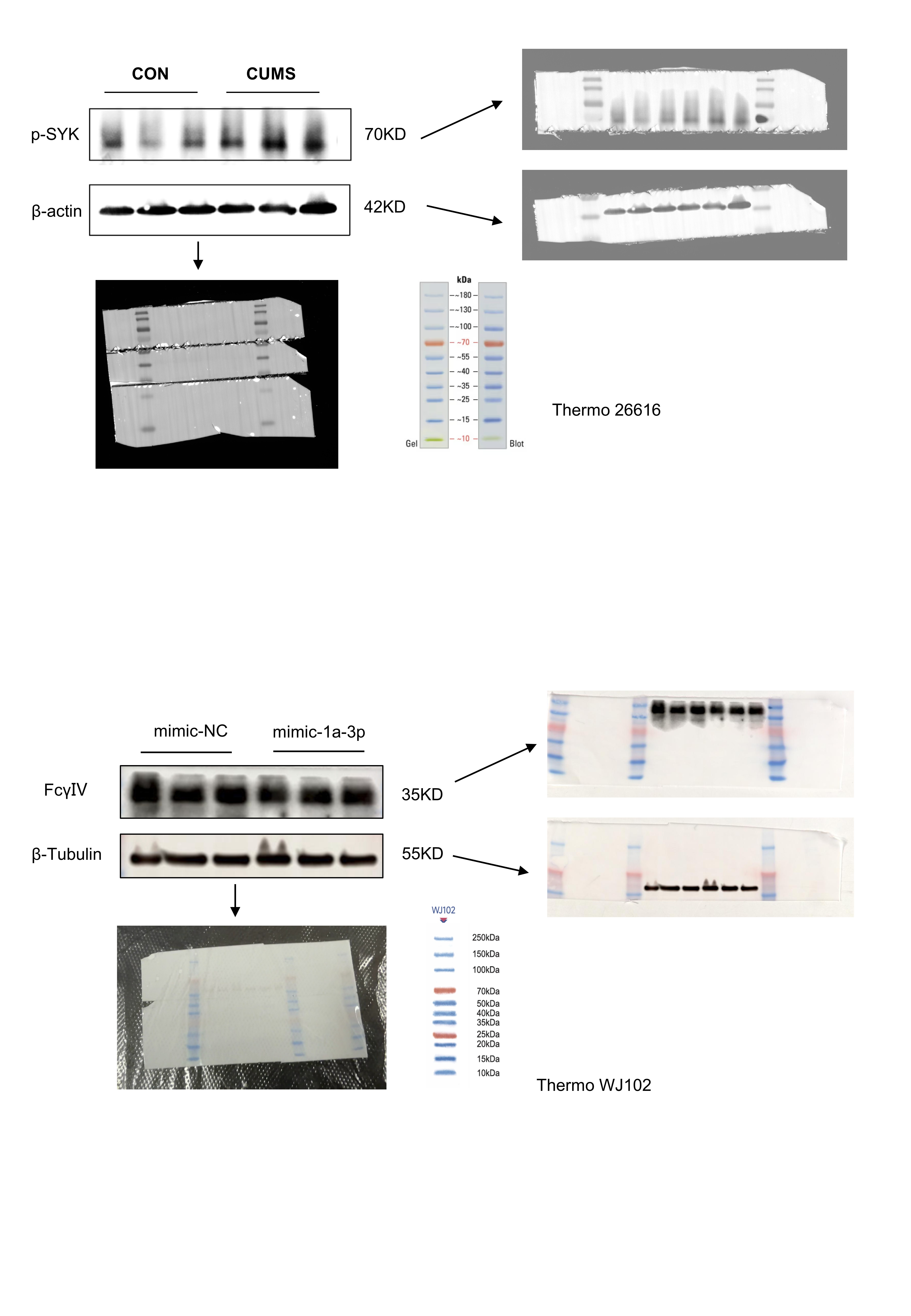


**Supplement table 1**

| Database | Gene | Log2FC | *p*-value |
| --- | --- | --- | --- |
| GSE93883 | hsa-miR-1-3p | 0.6973 | 0.036084256 |
| GSE221729 | mmu-miR-1a-3p | 0.4048 | 0.034712913 |
